# Addressing the healthcare challenge: introducing *Discover Health Systems*

**DOI:** 10.1007/s44250-022-00001-x

**Published:** 2022-08-03

**Authors:** Rodolfo Catena, Vanni Agnoletti, Fausto Catena

**Affiliations:** 1 Global Business School for Health, UCL, London, UK; 2 Department of Anaesthesia and Intensive Care, “M. Bufalini” Hospital, Cesena, Italy; 3 Department of General and Emergency Surgery, “M. Bufalini” Hospital, Cesena, Italy

It was in June 2021 when Vanni and Fausto contacted me with the idea for a new journal. In their role of healthcare leaders during the lockdown, they had developed new ideas to address the challenges the COVID-19 pandemic was posing to health systems. They soon realized, however, that finding a journal to publish this research was not an easy task. Unfortunately, academia often works in silos, and publishing research contributing to and building on multiple disciplines can be difficult. There were simply not many opportunities to publish new research on health systems improvement. As we were discussing this gap in the academic literature with other academics and health and social care professionals, we decided to write to Springer Nature to propose a new journal that would offer an opportunity for researchers and professionals who are working to improve the delivery of care.

Health expenditure in 2020 accounted for more than 12% of the GDP of countries such as Germany and the UK, for around 10% of the Italian GDP and for around 17% of the United States GDP in 2019 [1]. These numbers alone give us an idea on why improving health systems is essential. The long-term sustainability of accessible healthcare services is at stake in developing and developed countries. Health systems worldwide have, in front of them, the challenge of coping with increasing health expenditures and fewer resources available in their budgets.

The Sustainable Development Goals (SDGs) of the UN represent another rationale for this journal [2]. The third goal, ‘Ensure healthy lives and promote well-being for all at all ages’ [2], will require the improvement of health outcomes, which will be achievable only if health systems become more effective and more efficient. This journal welcomes research that can contribute to the achievement of the SDGs and, in general, that contributes to the improvement of health outcomes worldwide.

*Discover Health Systems* is open access to maximize the impact of research. Open access research is key to giving the same opportunities to rich and poor universities, rich and poor hospitals/healthcare organizations, and rich and poor health and social care practitioners/academics. More than ever, our goal is to decrease the gap in tools and resources between developed and developing countries.

The audience for this journal is diverse, and includes healthcare professionals (such as physicians, nurses, and social care workers), healthcare management and leadership, policymakers, and politicians, engineers, economists, IT and other professionals working with health systems research. Different professional and academic profiles need to contribute because of the complexity of the challenge. Research work that considers a range of tools and knowledge from different disciplines has the potential to identify new opportunities for improvement that have not been considered before. Different viewpoints such as the business and health policy perspectives are essential for addressing the challenge of health systems improvement successfully.

Qualitative, quantitative, and mixed-methods research will be accepted as we recognize the value of these different approaches to studying health systems. The unit of analysis will vary; for example, it can be the individual healthcare organization, the health/social care professional, or the health system itself. We accept full-length research articles, brief communications of empirical findings, reviews, perspectives, comments, case studies, registered reports, and data notes. The following is a list of the topics (not exhaustive) the journal will focus on:

- National health systems
- Private health systems
- Integration of health informatics into health systems
- Capacity building within health systems
- Funding of health systems
- Health policy
- Health outcomes—patients/staff
- Human resources
- Operations management
- Healthcare facility safety and quality (performance)
- Sustainability
- Disaster planning and response
- National-level training, accreditation, and regulation of healthcare workers or facilities
- Monitoring and performance of health systems
- Related research methodology
- Strategic management
- Digital health
- Healthcare finance
- Management of organizations
- Operating room and waiting list management
- Process management
- Value-Based Health Care.

Open access for all papers is made possible through an article-processing charge (APC). Discover Health Systems intends to maintain affordable APCs for researchers, and the current charges can be found here (https://www.springer.com/journal/44250/how-to-publish-with-us#Fees%20and%20Funding). Funding for this fee can be available (for more information please visit https://www.springernature.com/gp/open-research/funding), and certain institutions will have the APC covered by Springer Nature open access agreements (https://www.springernature.com/gp/open-research/institutional-agreements). Finally, the journal will also offer discounts and waivers of the APC in some cases not covered by the above—please contact the editorial office for further information.

Together with the journal, a public non-profit research centre, the CREAS (Centro di Ricerche e Studi nell’Ambito dei Sistemi Sanitari, https://www.creas-hrc.it/), has been created in Cesena, Italy, and a professional society will be launched to create a network of professionals working in this field. The research centre and society will provide opportunities for new projects to improve health systems and for events to share and spread new ideas and research. The CREAS research centre and the professional society will both contribute to the journal.

Today, we all face the question of how to improve health services worldwide. The Institute for Healthcare Improvement has identified six dimensions that health systems should focus on: safety, effectiveness, patient-centeredness, timeliness, efficiency, and equity [3]. The Value-Based framework focuses on patient outcomes and healthcare costs [4]. The current pandemic has taught us that humankind has an untapped potential to find solutions to complex problems when people focus on a common goal and collaborate together towards it. This journal aims to provide a common space for people to share their progress in tackling the challenges of health systems improvement worldwide.
